# SSR individual identification system construction and population genetics analysis for *Chamaecyparis formosensis*

**DOI:** 10.1038/s41598-022-07870-5

**Published:** 2022-03-08

**Authors:** Chiun-Jr Huang, Fang-Hua Chu, Yi-Shiang Huang, Yu-Ching Tu, Yu-Mei Hung, Yu-Hsin Tseng, Chang-En Pu, Cheng Te Hsu, Chi-Hsiang Chao, Yu-Shyang Chou, Shau-Chian Liu, Ya Ting You, Shuo-Yu Hsu, Hsiang-Chih Hsieh, Chieh-Ting Wang, Chi-Tsong Chen

**Affiliations:** 1 School of Forestry and Resource Conservation, National Taiwan University, Taipei, 10617 Taiwan; 2 Biodiversity Research Center, Academia Sinica, Taipei, 11529 Taiwan; 3 Department of Forensic Science, Investigation Bureau, Ministry of Justice, New Taipei City, 23149 Taiwan; 4 Institute of Biological Chemistry, Academia Sinica, Taipei, 11529 Taiwan; 5 Department of Life Sciences, National Chung Hsing University, Taichung, 402 Taiwan; 6 Hualien Forest District Office, Forestry Bureau, Council of Agriculture, Hualien, 97051 Taiwan; 7 Department of Applied Science, National Taitung University, Taitung, 95092 Taiwan; 8 The Experimental Forest, National Taiwan University, No. 12, Sec. 1, Qianshan Rd., Nantou County, 55750 Taiwan

**Keywords:** Biotechnology, Plant sciences

## Abstract

*Chamaecyparis formosensis* is an endemic species of Taiwan, threatened from intensive use and illegal felling. An individual identification system for *C. formosensis* is required to provide scientific evidence for court use and deter illegal felling. In this study, 36 polymorphic simple sequence repeat markers were developed. By applying up to 28 non-linked of the developed markers, it is calculated that the cumulative random probability of identity (*CP*_*I*_) is as low as 1.652 × 10^–12^, and the identifiable population size is up to 60 million, which is greater than the known *C. formosensis* population size in Taiwan. Biogeographical analysis data show that *C. formosensis* from four geographic areas belong to the same genetic population, which can be further divided into three clusters: SY (Eastern Taiwan), HV and GW (Northwestern Taiwan), and MM (Southwestern Taiwan). The developed system was applied to assess the provenance of samples with 88.44% accuracy rate and therefore can serve as a prescreening tool to reduce the range required for comparison. The system developed in this study is a potential crime-fighting tool against illegal felling.

## Introduction

Illegal logging is a severe problem in many timber-producing countries. Unplanned felling results in forest degradation, affects forest ecosystems and promotes the spread of pests and pathogens. In addition, large-scale deforestation causes forests to lose their soil and water conservation functions, thus leading to water shortages in the dry season and floods in the rainy season. These disasters brought by illegal or unplanned felling cause significant damages to the country.

Incidents of theft of valuable timber continue to occur, yet the crime investigations are complicated due to the lack of effective measures to present court evidence for conviction. To obtain the molecular evidence linking seized timber to illegally felled stumps, it is necessary to develop an individual identification system for tree species of high economic value, which are often the target of illegal felling.

*Chamaecyparis formosensis* Masam., also known as False Cypress, is endemic tree species in Taiwan distributed majorly in the cloud forest, a zonal forest type in the mid elevation (1700–2600 m) with extremely high biodiversity^1^. Gigantic *C. formosensis* is known for its superb timber quality. Due to its high quality and market value (4050 USD/m^3^, woodprice.forest.gov.tw), *C. formosensis* is critically threatened by illegal felling. Unplanned felling and poor management compromised the ecosystem and endangered these endemic species. Similar scenarios also happened to *Dalbergia* spp. (Leguminosae: Papilionoideae)^2,3^, *Fraxinus excelsior* (Oleaceae)^4^, *Swietenia macrophylla* (Meliaceae)^5^, and *Intsia palembanica* (Fabaceae)^6^. Moreover, timber production countries suffer from illegal felling, particularly in South-east-Asian, African and South American countries^7^. Although suspects were arrested on some occasions, lack of direct scientific evidence to link seized timber and stump had led to a failure of conviction in the majority^8^.

In recent years, countries suffering from serious illegal logging have successively begun to develop DNA-based timber individual identification systems that can provide court evidence for a conviction. The reported technology for individual identification systems includes DNA point difference-based technique SNP (Single Nucleotide Polymorphism)^9^, and DNA length difference-based techniques SSR (Simple Sequence Repeat)^4,6,8,10,11^, and INDEL(Insertion/Deletion). SNP, SSR, and INDEL are all co-dominant molecular markers classified into heterozygous and homozygous. Diversified types can be found at the same loci according to the pairwise characteristics of genes in the same allele. For example, human ABO blood type contains three genotypes: I^A^, I^B^, and i. When the genotype of the individual is I^A^I^B^, I^A^i, and I^B^i, it is a heterogeneous combination, showing blood types AB, A, and B respectively. When the genotype of the individual is I^A^I^A^, I^B^I^B^, ii, it is a homogenous combination, showing blood types A, B, and O, respectively. When reaching enough numbers of the polymorphic molecular markers, they can be used for individual identification and thus can be used to compare the seized timber to the illegally felled stumps. The reported individual identification systems have demonstrated their potentials to provide scientific evidence for court cases^4,6,8–11^.

The SSR individual identification technique has been developed for more than 30 years and has been widely used in DNA paternity testing, forensic examination, victim identification, and animal individual identification^12,13^. SSR marker is a co-dominant and highly reproducible DNA marker with the following characteristics: it has a high degree of polymorphism; it is abundant and evenly distributed in eukaryotic genome; most of them are not functional and can be efficiently and economically tested by PCR (polymerase chain reaction); last, the length is generally short which provides a higher opportunity to be amplified when applied to the lysed sample^13,14^. Therefore, SSR is the most commonly used method for individual identification systems^15,16^. To protect the cypress resources in Taiwan, we have developed and adapted several polymorphic SSR markers^17^ for individual identification.

In the illegal felling crime case reports *C. taiwanensis*^8^ and *F. excelsior*^4^, it is demonstrated that the SSR individual identification system can provide scientific evidence that is considered acceptable by court. In these cases, the individual identification system developed with genetic markers for those species were used to link seized timbers and victim trees, while considering the random probability of the same genotype appearing in the population. Therefore, convincible scientific proof with a confidence level close to 100% was accepted as court evidence for crime conviction.

The legality of wood products usually depends on their source^18^. SSR is also often used in genetic diversity and population structure analysis of species^14,19^, and can further predict species' geographic provenance and distribution. Genetic methods have been applied to confirm the source and trade routes of protected species^20,21^. However, filing the DNA of every individual is not feasible even if *C. formosensis* has been listed as an endangered species. Therefore, it is important to analyze the genetic variation and population structure of *C. formosensis*. Genotyping can reveal the provenance of the timber and greatly reduce the range of possible plant sources.

Due to the extensive planned logging and rampant illegal logging in the last century, *C. formosensis* has been listed as an endangered species by the IUCN Red Book (International Union for conservation of nature red list of threatened species). It has become one of the most concerning issues whether *C. formosensis* has lost its genetic diversity due to excessive logging. From conservation perspective, SSR marker can be used to find high population genetic diversity areas, to understand the local species’ genetic structure composition of these areas, and to further provide necessary conservation measures and management to these areas. SSR marker is an effective tool for studying the diversity of populations and providing harmonised standards (e.g. the number of allelic (*A*), observed heterozygosity (*Ho*), expected heterozygosity (*He*), the inbreeding coefficient (F_is_), the fixation index (F_st_)) for comparison with other species^22^.

The primary purpose of this study is to develop *C. formosensis* SSR individual identification system, which provides high discrimination power against genetic variation, in order to prevent the occurrence of illegal felling. Moreover, the biogeographical analysis of *C. formosensis* also facilitates providing provenance information of the seized timber. In addition to being a long-developed individual identification tool^15,16^, SSR is also often used in plant conservation and breeding^23,24^. The SSR markers developed in this study can also support the future *C. formosensis* afforestation by selecting mother trees with higher diversity.

## Result and discussion

### Development of new SSR markers for *C. formosensis*

**Figure 1:**
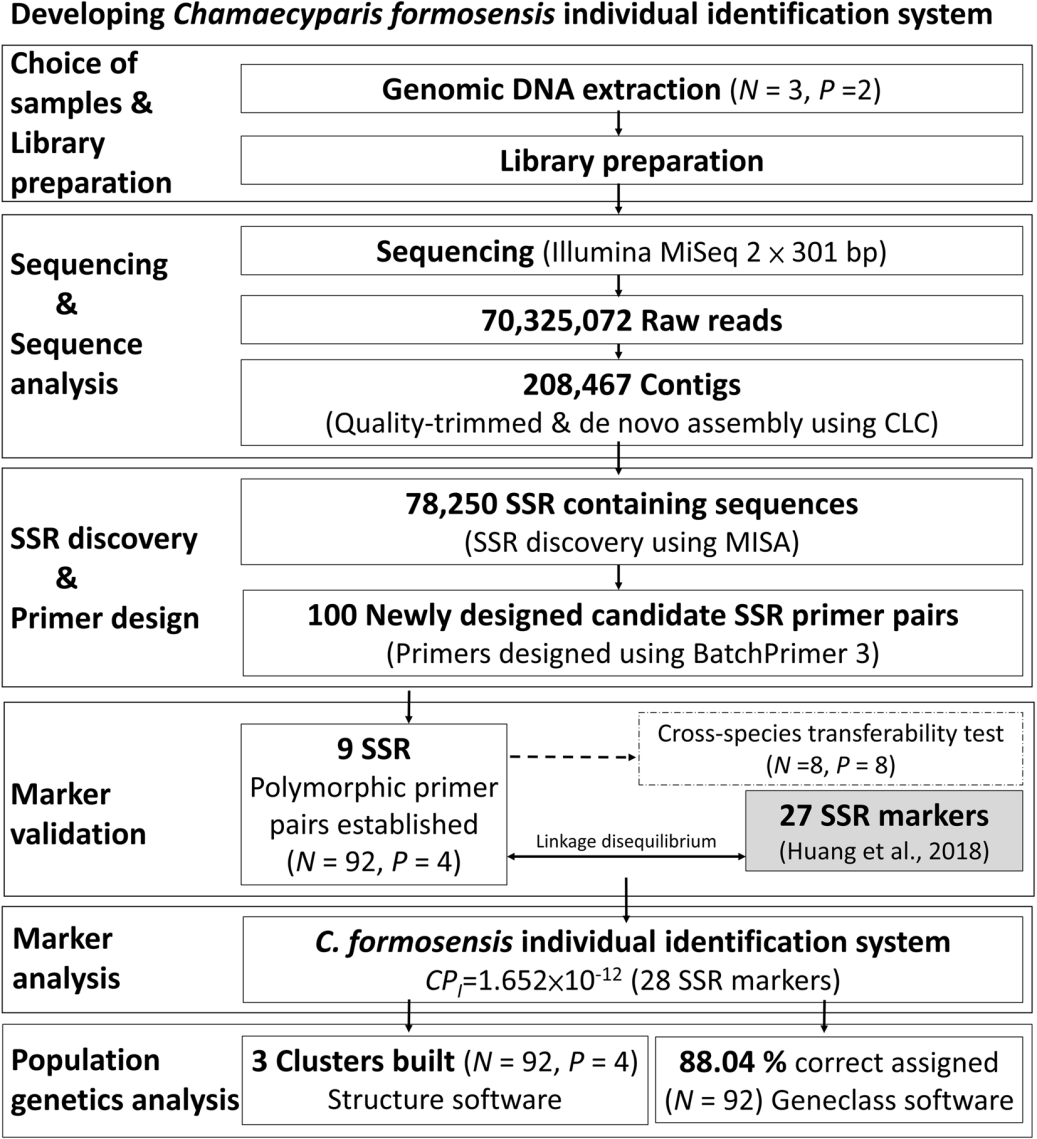
Flowchart of *Chamaecyparis formosensis* individual identification system development. *N*: the number of individuals; *P*: the number of populations; *MISA*: *Mi*cro*SA*tellite software; *CP*_*I*_ : combined probability of identity.

For a higher accuracy of court's judgment on illegal felling, it is necessary to establish a complete forensic system. Although some SSR markers of cypress have been published^17,25–27^, the reported detection rates for dried timber were only 20–40%^8,9^. Therefore, it is necessary to develop more SSR markers as a contingency plan. When the sample is in a poor condition, more markers can be applied in order to achieve the threshold of combined power of discrimination (*CPD*) required for successful comparison between seized timbers and victim trees. In order to maximize potential loci, Next generation sequence (NGS) methods were used. In this study 3 DNA library were constructed. We used the Illumina MiSeq platform (2 × 301 bp; Illumina, San Diego, California, USA) to sequence the DNA libraries (Fig. 1. and Supplementary 1.).

A total of 70,325,072 raw reads were produced. The raw reads were deposited in the NCBI BioProject (PRJNA454510). After quality-trimming to the raw reads with CLC Genomics Workbench version 10 (QIAGENE, Aarhus, Denmark), 70,319,509 contigs were generated with the length between 133 and 146 bp on average. De novo assembly was conducted with the following parameters: contig number 208,467, minimum length of contigs 18 bp, maximum length contigs 108,928 bp, and average length contigs 491 bp. The sequence was assembled with software CLC Genomics Workbench version 10, and the length of the assembly sequence was 102,281,642 bp.

A sum of 78,250 SSR containing sequences was screened by MISA (v 1.0, *Mi*cro*SA*tellite)^28^. We newly designed 100 candidate SSR primer pairs for testing in *C. formosensis* by BatchPrimer3^29^.

There are 9 validated SSR markers that are polymorphic (success rate 9.00%) were registered in GenBank in NCBI (Table 1) and passed for cross-species tests (Supplementary 2 and 3).

**Table 1:** Characteristics of 9 SSR loci developed in *Chamaecyparis formosensis*.

Locus	Primer sequences (5’ -3’)	Repeat motif	Fluorescent label	Allele size (bp)	*Ta* (°C)	GenBank accession no	Putative function [organism]
Cred603	TTGCTACATTAGCACTAGATAGCAAAGAAA	(AAG)13	6-FAM	106	60	MW052386	No hit
	ACTGAAGATACTGAGGATATTGAAGAGGAA						
Cred610	TGAGATATACATGTGTGAAAGAGAGTGAAGC	(GTAT)5	PET	166	60	MW052387	No hit
	TGCAATAATTTCTTCAGTGTTACCACTACC						
Cred628	GCTGGAGTCATTATAGTGCCATGTCTTTGT	(GCCC)3	6-FAM	142	60	MW052388	No hit
	TTTTCAAATAGCCGACCGACCTATGTAGAG						
Cred640	ACCCATATCTTCCTTCCCAACCATTAAGAT	(TCTT)5	6-FAM	137	60	MW052389	No hit
	CTTTCAGTGGAATGGAAGAAAGCCCTACTA						
Cred641	ACTTCTAATGAATCCCCATGCCGAATTGTA	(GC)19	VIC	193	60	MW052390	No hit
	CTGTTCGCGATAAGATAATTGGCTAGTGTG						
Cred674	TAAAGAGGCTCTGCTACTGGCTTTTCAACT	(GGGC)4	NED	147	60	MW052391	No hit
	GTGGGTGGCCCTCTATTCTATTGTTGAT						
Cred678	GGTCCATATCCTGGAGTAGAACCTCCCTAC	(GGGC)5	PET	162	60	MW052392	No hit
	GTGTCGCAGGCATAGACTTCTCCCTATATT						
Cred682	CCGCCCTTCTAATAACAGGGAAGATAAGTT	(CCCT)5	NED	147	60	MW052393	No hit
	CCGCCCTTCTAATAACAGGGAAGATAAGTT						
Cred683	GCAGCCTAAATAAACAATAGGGGGATTGAT	(GCCT)4	NED	146	60	MW052394	No hit
	CATGTTACGTATAGAATCGAGTGCAGGTCA						

Unlike the traditional SSR cloning method, with next-generation sequencing technology, it is easy to obtain a significant amount of SSR containing sequences from sequenced genomes^30^. However, transforming candidate SSR primer pairs into validated SSR markers is still a time-consuming and expensive step. Qualified SSR markers need to succeed in PCR amplification, have good peak pattern quality with minor stuttering, and be free of non-amplifying (invalid) alleles. The turnover rate from candidate SSR primer pairs to validated SSR markers varies from species to species^30,31^. The success rate in *Chamaecyparis* plants is between 5.24% and 9.27%^8,17,25,26,32^.

### Developing *C. formosensis* individual identification system

**Figure 2:**
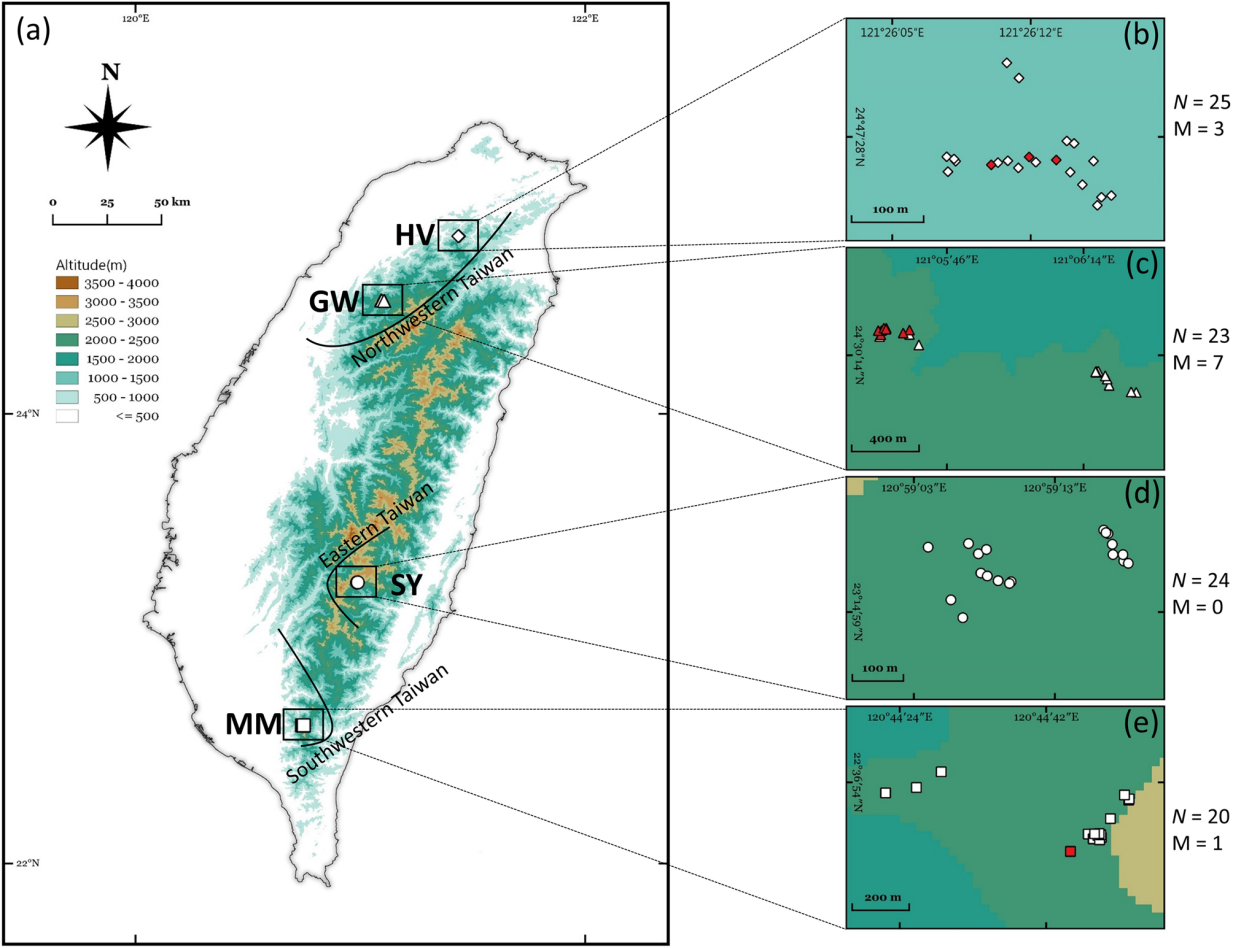
The biogeographic information of *Chamacyparis formosensis* in this study. A total of 92 samples composed of 20 MM, 25 HV, 23 GW, and 24 SY individuals were analyzed (**a**) Biogeographic analysis data suggests that the samples fall into three genetical categories: SY (Eastern Taiwan), HV & GW (Northwestern Taiwan), and MM (Southwestern Taiwan) (**b**)–(**e**) The red spots represent the individuals that have been mis-assigned (denoted as M in figure legend) from provenance simulation result. *N*: the number of individuals, *M*: the number of mis-assigned.

In this study, newly developed 9 validated SSR markers and other 27 validated SSR markers^17^ polymorphic SSR markers were analyzed against 92 individuals from 4 geographic areas (MM, HV, GW, SY, Fig. 2 and Supplementary 1). The results of developed 36 SSR markers are summarized in Table 2. Among the 92 individuals in this study, each number of alleles of SSR is between 2 and 27, with an average of 7.916. The levels of observed heterozygosity (*Ho*)^33^ are from 0.000 to 0.891, with an average of 0.414. The levels of expected heterozygosity (*He*)^33^ range from 0.103 to 0.906, with an average of 0.565. Significant (*P* < 0.001) deviations of Hardy–Weinberg equilibrium (HWE)^34^ were detected in 23 SSR loci: Cred47, 225, 231, 236, 242, 248, 249, 250, 253, 260, 262, 276, 277, 280, 603, 610, 628, 640, 641, 674, 678, 682, 683. *Ho* is the actual proportion of heterozygous individuals in each locus within the population, whereas the *He* is the expected value estimated per HWE. *Ho* and *He* are among the most widely used parameters in estimating genetic diversity in a population. The population structural and even historical information can be obtained from *Ho* and *He*. When *Ho* = *He*, it means that the population is random mating. When *Ho* < *He*, it means that the population is inbreeding. When *Ho* > *He*, it means that the population is outcrossing^33^. Most of these loci (36 tested) are *Ho* < *He* (except Cred211, Cred220, Cred225, Cred248, Cred276, Cred281, Cred297, Cred298), suggesting the population of *C. formosensis* has a low genetic divergence and is an inbred strain. HWE describes that under ideal conditions, there exists no mutations, no natural selection, no individuals moving in or out, the population is infinitely large, and random mating within the population. Therefore, gene frequency does not change over time or generation. However, there will always be one or more interfering factors (e.g. genetic drift, natural selection, mutation, gene flow, population bottleneck, founder effect, and inbreeding.) affecting gene frequency in nature^35^. Therefore, HWE is difficult to achieve in nature. In this study, 23 loci out of 36 markers deviated from HWE (63.89% deviation rate). The reason for this deviation could be artificial selection, non-panmixia or genetic drift.

**Table 2:**
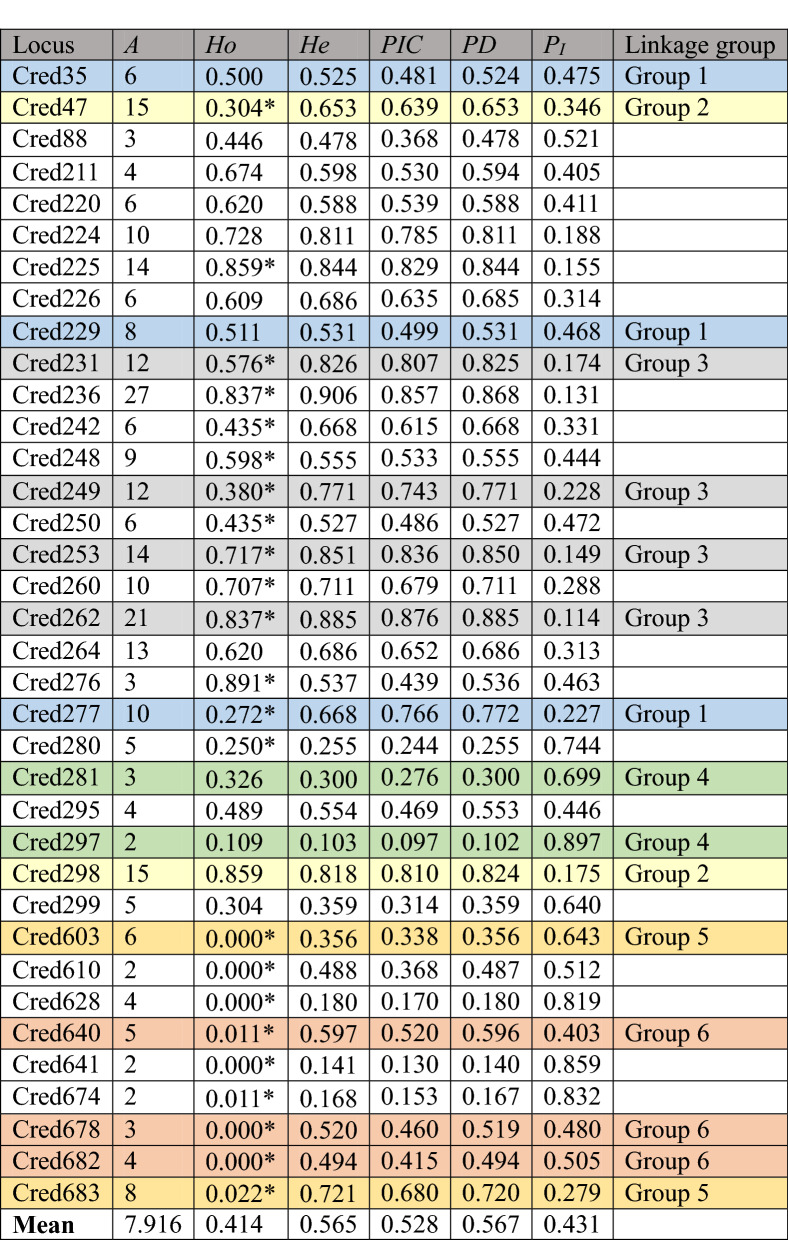
Genetic characterization of 36 polymorphic SSR loci of 92 *Chamaecyparis formosensis* individuals. *A:* number of alleles, *Ho:* observed heterozygosity, *He:* expected heterozygosity, *PIC:* polymorphism information content or power of information content, *PD:* power of discrimination, *P*_*I*_: the probability of identity, *PD* is equal to 1 – *P*_*I*_. *Highly significant from Hardy–Weinberg equilibrium (*P* < 0.001). Significant linkage disequilibrium (*P* < 0.001) was detected in the same colored pairs (Groups 1–6).

Polymorphism information content, or power of information content (*PIC*), is an index of the relative ability of the SSR marker's genetic variability. The higher the polymorphism of marker's genotype, the higher the *PIC* value^36^. Polymorphic markers were highly informative (*PIC* > 0.50), reasonably informative (0.50 > *PIC* > 0.25), and slightly informative (*PIC* < 0.25). Power of discrimination (*PD*)^37^ refers to the ability of genetic markers to distinguish individuals within a population. Obviously, in a population with more allele types and evenly distributed genotypes, the low probability of two random individuals having the same genotype, and the system can identify the greater probability of two random individuals. Probability of identity (*P*_*I*_)^38^ is the probability of two individuals with the same genotype. *PD* = 1-*P*_*I*_ . The value of *PIC*, *PD* and *P*_*I*_ of individual markers reflects its identification ability in the individual identification system. The greater *PIC* and *PD*, the lower *P*_*I*_ in value, suggesting the higher identification ability of the marker, and vice versa. The levels of *PIC* range from 0.097 to 0.876, with an average 0.528. The levels of *PD* range from 0.102 to 0.885, with an average 0.567. The levels of *P*_*I*_ range from 0.114 to 0.897, with an average 0.431. There were 19 out of 36 markers with *PIC* greater than 0.5, and the mean of these 36 markers *PIC* values was greater than 0.5, suggesting the markers have a high identification ability. The results of *PD* and *P*_*I*_ correspond to those of *PIC*. The highly informative markers presented in *PIC* also show higher identification ability in *PD* and *P*_*I*_.

Significant linkages (*P* < 0.001) were detected among Cred35/229/277 (Group 1), Cred47/298 (Group 2), Cred231/249/253/262 (Group 3), Cred281/297 (Group 4), Cred603/683 (Group 5) and Cred640/678/682 (Group 6) with GENEPOP 4.2^39^, suggesting the abovementioned group located in the same linkage group (Table 2). When identifying several independent polymorphic genetic markers simultaneously (polymorphic markers located in different linkage groups), the combined probability of identity (*CP*_*I*_) is the product of the *P*_*I*_ of each genetic marker. At this time, *CP*_*I*_ will be greatly reduced, and the combined power of discrimination (*CPD*) will become very high. As defined above, *CP*_*I*_ + *CPD* = 1. The credibility of the individual identification system is calculated based on "Random match probability in population size and confidence levels’" published by Budowle et al.^40^. Confidence levels (CL) = (1 – *CP*_*I*_)^*N*^, where *N* is number of individuals.

The individual identification system was applied to illegal felling cases^8^. When the seized timber and the victim tree are identified as the same particular plant, under the considerations of fairness and objective, the court usually adopts 99.99%, 99% or 95% confidence level as the credibility standard^41^, ISO ISO/IEC 17,025). In this study, the locus with the lowest *P*_I_ within a linkage group was used to calculate the *CP*_*I*_ (Table 3). The *CP*_*I*_ decreased along with accumulation of loci and the *P*_*I*_ of each locus were sorted in ascending order. The system can accumulate up to 28 loci without linkage. When reaching its maximum capability, even under most strict standard (confidence level 99.99%) dictated by court, this system can be used to identify 60 million *C. formosensis*, with *CP*_*I*_ as low as 1.652 × 10^–12^, *CPD* as high as 0.999999999998348 (almost equal to 1), beyond the known population size of 32.06 ± 3.20 million *C. formosensis*^42^. Under ideal conditions, a minimum of 6 loci can be applied to the system, with an identifiable *C. formosensis* population of 2,900 under 95% confidence level. The *CP*_*I*_ is as low as 1.728 × 10^–5^, and *CPD* is as high as 0.999982712603209 (Table 3).

**Table 3:**
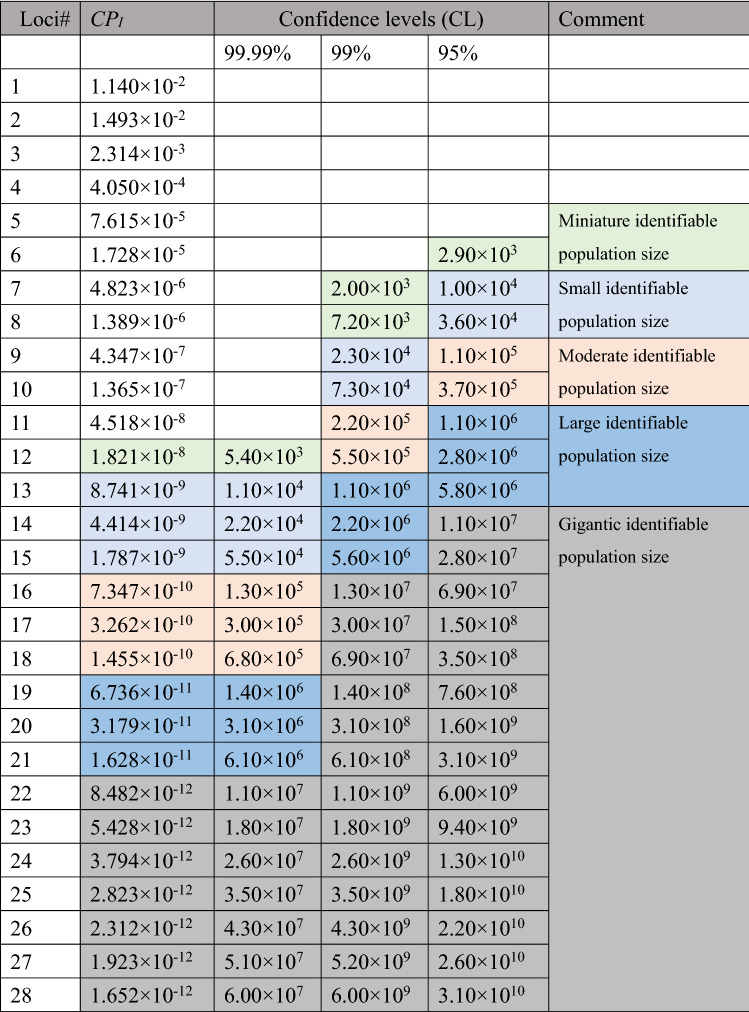
The discrimination power in SSR marker combination. *CP*_*I*_ cumulative random probability of identity, CL = (1- *CP*_*I*_)^*N*^, *N* number of individuals.

One of the problems with SSR marker is the appearance of null alleles. One possible cause of SSR null alleles is poor primer annealing caused by the nucleotide sequence divergence of the flanking primer on one or both sides (for example, point mutation or indel in the primer sequence)^43^. In addition, due to the competitive nature of PCR, smaller alleles usually have a higher amplification efficiency than larger alleles. Therefore, only the smaller of the two alleles can be detected from heterozygous individuals. The null alleles caused by differential amplification can usually be seen by loading more samples or adjusting the contrast^44^. The third cause of null alleles may be due to inconsistent quality or the low quantity of DNA templates. Some loci are relatively easy to amplify, yet others cannot be amplified within the same DNA preparation^45^. When a null allele is present, the observed genotype represents one of the several possible true genotypes^46^. SSR markers inevitably produce null alleles, and each SSR marker has a different background for null alleles.

Dakin et al. (2004)^44^ reviewed 233 publications by examining how authors detect and deal with null alleles and the methods used to estimate the frequency of null alleles across articles. The authors demonstrate that the frequency of simulated null alleles is usually overestimated, which will lead to underestimating the usability of this marker. It was misunderstood that the existence of null alleles will reduce the availability of paternity testing, individual identification, and population genetic research. However, it has been demonstrated that null alleles do not change the overall result on assignment testing^43,44^. Compared with the presence of null alleles, increasing the number of loci and the degree of genetic differentiation has a more significant impact on the accuracy of assignment testing. This argument is valuable for studying SSR markers and populations prone to invalid alleles, as it allows researchers to use loci affected by invalid alleles^43,44^.

In Huang et al.^8^ where *C. taiwanensis* individual identification system was applied to an illegal felling conviction case, CPD calculations exclude any markers that show homozygous PCR results per ISO/IEC 17,025. The CPDs are calculated only from the possibilities of the markers found in timber and tree samples simultaneously. Null alleles and PCR fail will only reduce the identification rate but will not cause seized timber and victim tree from different individuals to be identified as the same source. However, this is not to say that efforts should not be made to use loci that display low-frequency null alleles. On the contrary, markers that are less prone to invalid alleles should always be preferred because they are less ambiguous and are more potent in assignment testing. However, before many individual identification markers are developed and optimised, the impact of null alleles should not be overemphasized, as it reduces the usability of markers^43,44^.

### Population genetics analysis

F_is_, by definition F_is_ = 1-*Ho*/*He*, is the inbreeding coefficient of an individual concerning the local subpopulation. When *Ho* < *He*, then F_is_ > 0, indicating that the population is an inbreeding. The 36 polymorphic SSRs were used to evaluate the genetic diversity parameters of the four groups (MM, HV, GW, SY) (Table 4). The number of alleles (*A*) for each locus is 4.417 and 5.444. *Ho* and *He* are ranged from 0.376 to 0.506 and from 0.474 to 0.583, respectively. All the groups show positive inbreeding coefficients, suggesting these four groups are inbreeding lines.

**Table 4:** Detailed genetic diversity parameters were identified at 36 simple sequence repeat (SSR) in four populations (MM, HV, GW, SY) of *Chamaecyparis formosensis. N*: the number of individuals, *A*: number of alleles, *Ho*: observed heterozygosity, *He*: expected heterozygosity, F_is_: inbreeding coefficient. When *Ho* < *He*, F_is_ > 0, indicating that the population is inbreeding^67^.

Population	*A*	*Ho*	*He*	F_is_
MM (*N* = 20)	4.417	0.376	0.474	0.206
HV (*N* = 25)	4.417	0.390	0.508	0.231
GW (*N* = 23)	5.444	0.380	0.503	0.242
SY (*N* = 24)	5.278	0.506	0.583	0.132
Average	4.889	0.413	0.517	0.200

The fixation index (F_st_) estimates population differentiation due to genetic structure^47^. A higher F_st_ value means a higher degree of difference between populations. When F_st_ is less than 0.05, there is no differentiation among populations. When F_st_ is between 0.05 and 0.15, there is low differentiation among populations. On the other hand, the estimation of the number of migrants (Nm) is gene flow value^47^. If Nm is more than one, genes frequently exchange, which counteracts the genetic drift and prevents the population differentiation^48^. If Nm is greater than four, the population is a random mating^49^. The analyses of F_st_ and Nm of the four geographic areas were conducted by GeneAlex 6.503^50^ (Table 5). The F_st_ value between HV and GW was 0.035, suggesting no population differentiation in these two populations. The highest F_st_ value (0.074) was found between HV and MM. The F_st_ values ranged from 0.056 to 0.065 were found between the rest geographic areas, indicating a low differentiation in these geographic areas. The highest Nm value (6.832) was found between HV and GW, whereas the lowest value (3.141) was between HV and MM. The Nm values of four geographic areas were greater than 1 (between 3.141 and 6.832), suggesting a frequent gene exchange between the four geographic regions, which offsets genetic drift and prevents population differentiation. For GW/MM (Nm = 4.022), GW/HV (Nm = 6.382), the Nm values of the population are more significant than four, suggesting that these populations are random mating.

**Table 5:** Pairwise F_st_ and Nm among four populations (MM, HV, GW, SY) of *Chamaecyparis formosensis* using 36 simple sequence repeat (SSR) data. *N:* the number of individuals; F_st_: the fixation index. F_st_ < 0.05, no differentiation among populations. 0.05 < F_st_ < 0.15, low differentiation among populations. Nm: the gene flow value. Nm > 1 represents the frequent exchange of genes, which counteracts the genetic drift and prevents the population differentiation^48^. When Nm is greater than 4, it would be a random mating population^49^.

Nm	F_st_			
	MM (*N* = 20)	HV (*N* = 25)	GW (*N* = 23)	SY (*N* = 24)
MM (*N* = 20)	–	3.141	4.022	3.798
HV (*N* = 25)	0.074	–	6.832	3.603
GW (*N* = 23)	0.059	0.035	–	4.199
SY (*N* = 24)	0.062	0.065	0.056	–

**Figure 3:**
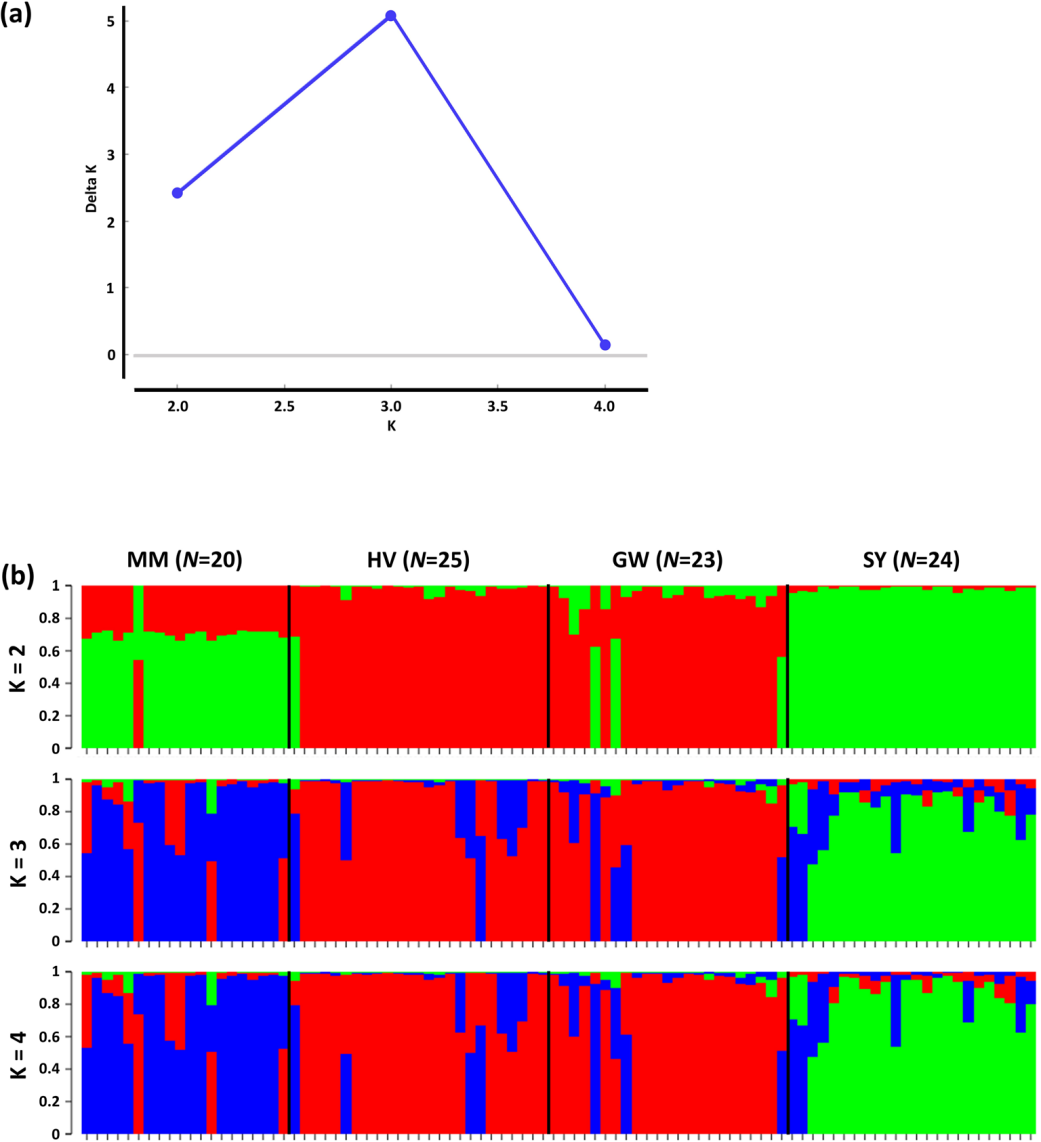
Genetic composition of *Chamaecyparis formosensis*. (**a**) The scatter plots of Delta K. (**b**) The 2, 3 and 4 clusters obtained from STRUCTURE analyses.

STRUCTURE analysis^51,52^ was used to analyze the population genetic structure of *C. formosensis* (Fig. 3), and the Delta K value was calculated to obtain the optimal number of clusters. K and Delta K are shown in Fig. 3a. The individuals of *C. formosensis* were most likely to be three clusters (Fig. 3b): the SY located in Eastern Taiwan is an independent cluster, the MM located in Southwestern Taiwan is another cluster, whereas the two HV and GW geographic areas are in the same genetic cluster. The results of F_st_ (Table 5), Nm (Table 5) and STRUCTURE analyses (Fig. 3) show that *C. formosensis* of the four geographical areas belongs to the same genetic population. The F_st_ and STRUCTURE analyses suggest that the samples fall into three clusters. The hypothesis that Taiwan Island is one of the plant refuges during the Quaternary glaciation^53,54^ may help to explain the results. The study of historical biogeography and phylogeny of cypress^54^ suggested that *C. formosensis* in Taiwan diverged from *Chamaecyparis* in Japan 2.9 million years ago. The arrival of the Quaternary glaciation led to species extinction and the continued retreat of species to lower latitudes^55^; thus Taiwan Island became a refuge for many ancient species, such as *Juniperus morrisonicola* (Cupressaceae)^56^, *Abies kawakamii* (Pinaceae)^57^, *Castanopsis carlesii* (Fagaceae)^58^. After the glaciation, species spread from the refuge to the surrounding areas and created species diversity across the latitude gradient^59^. In our results, the low polymorphism of *C. formosensis* probably indicates that they originally derived from the same large population during the glaciation. After the glacial retreat, these four *C. formosensis* clusters spread out from the refuge and formed the four populations due to geographic isolation.

Studies^55,60^ also show that, based on molecular evidence, many plants (eg. *Cunninghamia konishii*, *Cyclobalanopsis glauca*, *Trochodendron aralioides*) in Taiwan island have high genetic diversity, higher than that of mainland China and Japanese archipelago. This remarkable high genetic diversity is associated with the Ice Age history in Taiwan^55,60^. The low genetic diversity of *C. formosensis* differs from most Taiwanese plants but is similar to another endangered plant of the genus *Cypress*, *C. taiwanensis*, in Taiwan Island (*A* = 6.507, *Ho* = 0.392, *He* = 0.501)^8^. Compared to *C. obtusa*, an endangered cypress plant in the Japanese archipelago, *C. formosensis* is also inbreeding (F_is_ = 0.034), but the degree of genetic diversity (*A* = 23.9) is significantly lower. One possible explanation of the low genetic diversity is that a large population of *C. formosensis* was divided into several smaller populations after ancient glacial retreat in Taiwan, and then they were recently overexploited by humans (REF).

GENECLASS v. 2.0^61^ was applied to analyse the provenance of 92 individuals independently. The probability of samples returning to the correct provenance is 95.00% (MM), 88.00% (HV), 69.57% (GW), and 100.00% (SY), with an overall mean correct rate of 88.04% (Table 6). Three HV individuals were misassigned to GW and four GW individuals were misassigned to HV, corresponding to the observation that HV and GW are the same clusters. However, three GW individuals were misassigned to MM, possibly because the geographic location of GW is between HV and MM. Therefore, GW has characteristics of north and south at the same time. Likelihood, one MM was mis-assigned to GW, further supporting the inference that there is partial gene exchange between MM and GW. Our data show that the populations in eastern (SY) and western Taiwan (the rest populations) have distinct genotype differences. Within the western populations, the northern (HV) and the southern ones (MM) have obvious differences. Therefore, when seizing timbers in the future, the genotype can be served as a prefilter to infer the geographic area of the victim tree if the provenance is found to be MM, HV or SY. A further inspection is required if the provenance is GW because of the existence of gene exchange between nearby geographic areas.

**Table 6:** *Chamaecyparis formosensis* individual provenance simulation result. A total of 92 samples composed of 20 MM, 25 HV, 23 GW, and 24 SY individuals were subjected to provenance simulation. Correct provenance is 95.00% (MM), 88.00% (HV), 69.57% (GW), and 100.00% (SY), with an overall mean correct rate of 88.04%.

	MM (*N* = 20)	HV (*N* = 25)	GW (*N* = 23)	SY (*N* = 24)	Over all (*N* = 92)
Sum of correct samples	19	22	16	24	81
Correct cluster (%)	95.00	88.00	69.57	100.00	88.04

## Conclusions

In this study, a *C. formosensis* individual identification system was built with 36 polymorphic SSR markers. When 28 non-linked SSR markers are applied, the system is capable of identifying 60 million *C. formosensis* individuals with a confidence level of 99.99%. The lowest *CP*_*I*_ is 1.652 × 10^–12^, and the highest *CPD* is 0.999999999998348. This system can provide the scientific evidence to link seized timbers and victim trees required the illegal felling court cases and facilitate future legal sales by profiling timbers. Through population genetics analysis, the system can provide provenance information, which would significantly enhance the efficiency by reducing the range required for investigation. The polymorphic markers developed in this study can be further applied to the conservation and breeding of the endangered species *C. formosensis*.

## Materials and methods

### Development of new SSR markers for *C. formosensis*

In order to develop SSR markers for individual identification, we constructed three DNA libraries. Three *C. formosensis* individuals from QL (Voucher no. *Chung 4450*) and SY (Voucher no. *Chung 4905, 4906*) were used for DNA library preparation. To build three DNA libraries, genomic DNA was extracted from fresh leaves using the VIOGENE plant DNA extraction kit (VIOGENE, New Taipei City, Taiwan). The DNA libraries were sequenced using the Illumina MiSeq System (2 × 301 bp paired-end; Illumin, San Diego, California, USA) at Tri-I Biotech (New Taipei City, Taiwan).

Bioinformatics analysis was conducted with CLC Genomics Workbench version 10 (QIAGENE, Aarhus, Denmark). The raw reads were prescreened to remove adapter sequences and reads with greater than 0.01 error or an average quality less than QV20. The trimmed sequences were further subjected to de novo assembly.

MISA (*MI*cro*SA*tellite v 1.0)^28^ was applied to screen the SSR containing sequences from contigs. To design SSR primers, sequences with at least five di-, tri-, tetra-, penta-, and hexa-nucleotide repeats were selected using BatchPrimer3^29^, with optimized conditions set length at 18–23 bp, melting temperature 45–62 °C, and product size of 80–300 bp.

A total of 100 candidate SSR primer pairs were newly designed in this study. These markers were subjected to validation test on 92 samples from four *C. formosenses* geographic areas (MM, HV, GW, SY see Supplementary 1). The samples DNA used in marker validation were extracted using the VIOGENE plant DNA extraction kit (VIOGENE, New Taipei City, Taiwan). The PCR reaction was conducted with a final volume 20 μL containing 2 ng of genomic DNA, 0.25 μL of 10 μM each primer and 10 μL of Q-Amp 2 × Screening Fire Taq Master Mix (Bio-Genesis Technologies, Taipei, Taiwan). The following PCR process was conducted: an initial denaturation of 95 °C for 2 min; 30 cycles of 95 °C for 45 s, a primer-specific annealing temperature for 45 s, and 72 °C for 45 s; followed by a 15-min extension at 72 °C (Table 1). The amplified products were evaluated on the ABI 3500 (Applied Biosystems, Waltham, Massachusetts, USA) with GeneScan 600 LIZ Size Standard (Applied Biosystems). Fragment size was determined by using GeneMapper ID-X v1.6 (Applied Biosystems). The capillary electrophoresis diagrams for genotyping are shown in Supplementary 4.

The cross-species transferability of the designed markers was tested in *Chamaecyparis taiwanensis*; for details, see Supplementary 2 and 3.

### Developing *C. formosensis* individual identification system

Marker analysis was conducted by combining 27 pairs published SSR markers^17^ with 9 validated SSR markers abovementioned. GenAlex 6.51b2^50^ was used to calculate number of alleles (*A*), observed heterozygosity (*Ho*), expected heterozygosity (*He*), Hardy–Weinberg equilibrium (HWE). PowerMarker V3.25^62^ was used to calculate polymorphism information content or power of information content (*PIC*)^63^. Power of discrimination (*PD*)^37^, *PD* = 1 − Σ*P*_*i*_^2^, where *P*_*i*_ is the frequency of genotype *i* . Probability of identity (*P*_*I*_)^38^, *P*_*I*_ = 1 − PD. The combined power of discrimination (*CPD*)^37^, here we calculated *CPD* of 28 markers. *CPD* = 1 – [(1 – *PD*_*1*_)(1 – *PD*_*2*_)…(1 – *PD*_*28*_)].The combined probability of identity (*CP*_*I*_)^38^. Microsoft Excel (Microsoft Office 2016) was used to calculate *PD*, *P*_*I*_, *CPD*, *CP*_*I*_. GENEPOP 4.2^39^ was used to test for linkage disequilibrium.

### Population genetics analysis

Genetic diversity parameters, genetic differentiation and gene flow among 4 geographic areas (MM, HV, GW, SY) were analyzed using F_is_, F_st_ and Nm by GenAlex 6.503^50^.

The population genetic structure was analyzed using STRUCTURE 2.3.4^52^. The program was run for K = 1 to 5 clusters with 20 independent runs to assess simulation stability. Each simulation was run for an initial 1,000,000 burn-in period followed by 100,000 replications based on the Markov chain Monte Carlo (MCMC)^64^. The best grouping was evaluated by Delta K^64^ in Structure Harvester Web v0.6.94^65^. Bar graphs were generated by CLUMPP 1.1.2^66^ for K ideal.

Individual provenance simulation was conducted with GENECLASS v. 2.0^61^ on every 92 individuals independently. A pairwise simulation was also conducted on the pooled database deducted the sample itself.

### Plant collecting permit declaration

With legislation compliance of experimental materials, we hereby declare that all of our experimental research and field studies on plants, either cultivated or wild, including the collection of plant material, comply with relevant institutional, national, and international guidelines and legislation.

### Software and data use declaration

In this research, the software and the data generated by the software (including commercial software, open-licensed software) are used legally in accordance with regulations, which allow to reproduction, distribution, transmit and modification works (including commercial use).

## Supplementary Information

Supplementary Information.

## Data Availability

Raw sequence information and developed SSR primer pairs have been deposited to NCBI (BioProject ID PRJNA454510); GenBank accession numbers are provided in Table 1.
