# Supplementary material for: SSR individual identification system construction and population genetics analysis for Chamaecyparis formosensis
Source: Sci Rep. 2022 Mar 8;12:4126. doi: 10.1038/s41598-022-07870-5 (PMC8904461; doi:10.1038/s41598-022-07870-5)

**SSR individual identification system construction and population genetics analysis for**

***Chamaecyparis formosensis***

Chiun-Jr Huang^1,2,3*^, Fang-Hua Chu^1^, Yi-Shiang Huang^4^, Yu-Ching Tu^3^, Yu-Mei Hung^3^, Yu-Hsin Tseng^5^, Chang-En Pu^3^, Cheng Te Hsu^6^, Chi-Hsiang Chao^3^, Yu-Shyang Chou^3^, Shau-Chian Liu^7^, Ya Ting You^2^, Shuo-Yu Hsu^1^, Hsiang-Chih Hsieh^1^, Chieh-Ting Wang^8^ and Chi-Tsong Chen^3*^

1. School of Forestry and Resource Conservation, National Taiwan University, Taipei 10617, Taiwan.
2. Biodiversity Research Center, Academia Sinica, Taipei 11529, Taiwan.
3. Department of Forensic Science, Investigation Bureau, Ministry of Justice, New Taipei City 23149, Taiwan.
4. Institute of Biological Chemistry, Academia Sinica, Taipei 11529, Taiwan.
5. Department of Life Sciences, National Chung Hsing University, Taichung 402, Taiwan
6. Hualien Forest District Office, Forestry Bureau, Council of Agriculture, Hualien 97051, Taiwan.
7. Department of Applied Science, National Taitung University, Taitung 95092, Taiwan.
8. The Experimental Forest, National Taiwan University, No. 12, Sec. 1, Qianshan Rd., Nantou County, Taiwan 55750, Taiwan.

***Corresponding authors:** [d04625001@ntu.edu.tw](mailto:d04625001@ntu.edu.tw); [chen33039@gmail.com](mailto:chen33039@gmail.com)

**Supplementary 1.** List of voucher information of the 3 NGS libraries and 4 populations where samples were collected and used to develop the 9 SSR markers in this study. All voucher specimens are deposited in the Herbarium of the Biodiversity Research Center (HAST), Academia Sinica, Taipei, Taiwan.

| Voucher no. | Collection Locality | Geographic coordinates | Population Code | *N* |
| --- | --- | --- | --- | --- |
| *Chung 4450* | Qilan Forest Recration Area, Datong Township, Yilan Country 267, Taiwan. | 24°35'26.28''N,  121°26'15.34''E | QL | 1 |
| *Chung 4905, 4906* | Siangyang Forest Recreation Area, Haiduan Township, Taitung Country 957, Taiwan. | 23°15'1.40''N,  120°59'8.54''E | SY | 1 |
| *Chung 3143, 3144, 3145, 3158, 3161, 3165, 3167, 3168, 3169, 3170, 3171, 3172, 3174, 3177, 3178, 3180, 3181, 3182, 3183, 3184* | Meli-miligang, Taiwu Township, Pingtung County 921, Taiwan. | 22°36'53.37''N,  120°44'26.05''E | MM | 20 |
| *Chung 3185, 3186, 3187, 3188, 3189, 3192, 3193, 3194, 3195, 3196, 3198, 3201, 3202, 3203, 3204, 3205, 3206, 3210, 3211, 3212, 3214, 3215, 3216, 3217, 3218* | Herve Divine Trees, Fuxing Dist, Taoyuan City 336, Taiwan. | 24°47'27.13''N,  121°26'14.86''E | HV | 25 |
| *Chung 4007, 4008, 4009, 4010, 4011, 4012, 4013, 4015, 4016, 4017, 4018, 4022, 4023, 4025, 4026, 4027, 4028, 4030, 4031, 4032, 4033, 4034, 4035* | Guanwu Forest Recreation Area, Tai’ an Township, Miaoli County 365, Taiwan. | 24°30'6.18''N,  121°05'30.66''E | GW | 23 |
| *Chung 4254, 4255, 4256, 4257, 4258, 4259, 4260, 4261, 4262, 4263, 4264, 4265, 4266, 4268, 4269, 4277, 4281, 4282, 4284, 4285, 4286, 4287, 4288, 4289* | Siangyang Forest Recreation Area, Haiduan Township, Taitung Country 957, Taiwan. | 23°15'1.40''N,  120°59'8.54''E | SY | 20 |

**Supplementary 2.** List of *Chamaecyparis taiwanensis* individuals used in cross-species transferability test on the 9 validated SSR markers.

| Voucher no. | Collection Locality | Geographic coordinates | Population Code |
| --- | --- | --- | --- |
| *Chung 2435* | Taipingshan Forest Recreation Area, Datong Township, Yilan County 267, Taiwan. | 24°29'40.96''N,  121°32'6.59''E | TP |
| *Chung 2476* | Cueifong Lake, Datong Township, Yilan County 267, Taiwan. | 24°30'37.45''N,  121°36'32.52''E | CF |
| *Chung 3116* | No.7 provincial highway, Yilan City, Yilan County 260, Taiwan. | 24°38'40.08''N,  121°26'39.47''E | NC |
| *Chung 3241* | Dasyueshan Forest Recreation Area, Heping Dist, Taichung City 424, Taiwan. | 24°13'9.59''N,  120°53'9.06''E | DS |
| *Chung 4021* | Guanwu Forest Recreation Area, Tai’ an Township, Miaoli County 365, Taiwan. | 24°30'6.18''N,  121°05'30.66''E | GW |
| *Chung 4190* | Lalashan Forest Recreation Area, Fuxing Dist, Taoyuan City 336, Taiwan. | 24°32'17.04''N,  121°17'40.03''E | LL |
| *Chung 4432* | Qilan Forest Recration Area, Datong Township, Yilan Country 267, Taiwan. | 24°35'26.28''N,  121°26'15.34''E | QL |
| *Chung 4541* | No.160 Forest Road, Jianshi Township, Hsinchu County 313, Taiwan. | 24°32'22.04''N,  121°22'40.74''E | FR |
| *Chung 2435* | Taipingshan Forest Recreation Area, Datong Township, Yilan County 267, Taiwan. | 24°29'40.96''N,  121°32'6.59''E | TP |

**Supplementary 3.** The cross-species transferability test of 9 validated SSR markers was performed with *Chamaecyparis taiwanensis* from 8 populations. Plus sign: marker has PCR product(s); Minus sign: marker has no PCR product.

| Locus | TP | CF | NC | DS | GW | LL | QL | FR | +/- |
| --- | --- | --- | --- | --- | --- | --- | --- | --- | --- |
| Cred603 | + | + | + | + | + | + | + | + | 8/0 |
| Cred610 | + | - | - | - | - | - | - | - | 1/7 |
| Cred628 | + | + | + | + | + | + | + | + | 8/0 |
| Cred640 | - | - | - | - | - | - | - | - | 0/8 |
| Cred641 | + | - | - | - | - | - | - | - | 1/7 |
| Cred674 | + | + | + | + | + | + | + | + | 8/0 |
| Cred678 | + | + | + | + | + | + | + | + | 8/0 |
| Cred682 | + | - | - | - | - | - | - | - | 1/7 |
| Cred683 | + | - | - | - | - | - | - | - | 1/7 |

**Supplementary 4.** Capillary electrophoresis diagrams for genotyping. SSR marker is a co-dominant molecular marker. For a heterogeneous sample, two peaks can be found in the diagram. The type marks for upper-left and lower-left samples are Sample 3144 (125, 147) and Sample 4008 (143, 147). Only one peak can be found in the diagram for a homogeneous sample. The type marks for upper-right and lower-right samples are Sample 3145 (143, 143) and Sample 3161 (147, 147).


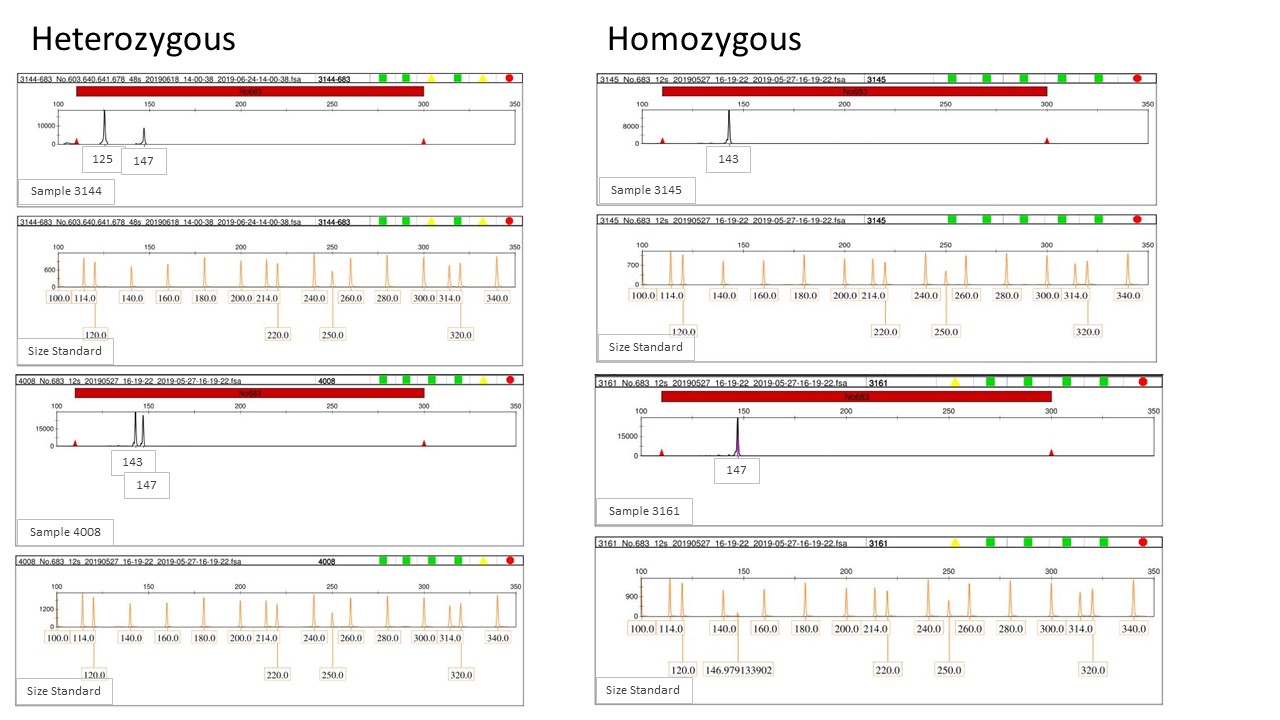

Supplement: Supplementary file 1 — Supplementary Information. [file 41598_2022_7870_MOESM1_ESM.docx]
